# Enhancing Health and Public Health through Machine Learning: Decision Support for Smarter Choices

**DOI:** 10.3390/bioengineering10070792

**Published:** 2023-07-02

**Authors:** Pedro Miguel Rodrigues, João Paulo Madeiro, João Alexandre Lobo Marques

**Affiliations:** 1 CBQF—Centro de Biotecnologia e Química Fina—Laboratório Associado, Escola Superior de Biotecnologia, Universidade Católica Portuguesa, Rua de Diogo Botelho 1327, 4169-005 Porto, Portugal; 2 Department of Computing, Federal University of Ceará, Fortaleza 60440-900, Ceará, Brazil; jpaulo.vale@dc.ufc.br; 3 Laboratory of Applied Neurosciences, University of Saint Joseph, Macao SAR 999078, China; alexandre.lobo@usj.edu.mo

In recent years, the integration of Machine Learning (ML) techniques in the field of healthcare and public health has emerged as a powerful tool for improving decision-making processes. The ability of ML algorithms to analyze vast amounts of data, identify patterns, and generate actionable insights has opened new avenues for enhancing various aspects of healthcare delivery and public health initiatives. This Special Issue (SI) explores the applications of ML in health and public health decision support systems, highlighting their potential benefits and challenges, mainly in the following areas:

1. Disease Diagnosis and Prognosis—In this area, ML algorithms can analyze patient data, including medical records, lab results, and imaging scans, to aid in the diagnosis and prognosis of various diseases. By training on large datasets, these algorithms can learn to recognize patterns and make accurate predictions, helping healthcare professionals make informed decisions about treatment plans and interventions. ML models have shown promising results in detecting conditions such as cancer [1], cardiovascular diseases [2], neurological diseases [3], and infectious diseases [4], enabling early detection and timely interventions. In this sub-area of study, the SI contributes with the following studies:
  - Mirniaharikandehei et al. [5] explore the feasibility of using a modified deep learning (DL) method for automatically segmenting disease-infected regions and predicting disease severity in computed tomography (CT) images. A dataset from 20 COVID-19 patients has been used, incorporating manually annotated lung and infection masks. An ensemble DL model was trained, combining five customized residual attention U-Net models for disease-infected region segmentation and a Feature Pyramid Network model for disease severity stage prediction. The analysis reveals >90% agreement in disease severity classification between the DL model and radiologists for 45 testing cases.
  - Chen et al. [6] explore a noninvasive, cost-effective tool to assess the risk of subclinical renal damage (SRD) in asymptomatic individuals. Using ML algorithms, a risk assessment score model was established based on systolic blood pressure, diastolic blood pressure, and body mass index. The model demonstrated excellent classification ability, with an AUC value of 0.778 for SRD estimation and 0.729 for 4-year SRD risk prediction.
  - Zhang et al. [7] investigate the effects of atherosclerotic intracranial internal carotid artery stenosis (IICAS) on extracranial internal carotid artery (ICA) flow velocity waveforms to identify sensitive hemodynamic indices for IICAS diagnoses. Hemodynamic indices, including peak systolic velocity (PSV), end-diastolic velocity (EDV), resistive index (RI), and the first harmonic ratio (FHR), were analyzed in simulations with and without IICAS. In a case-control study with patients having mild-to-moderate IICAS, statistical analyses revealed that the average PSV, EDV, and RI were lower in the stenosis group compared to the control group, but without significant differences (*p* > 0.05), except for the PSV of the right ICA (*p* = 0.011). However, the FHR showed a significantly higher value in the stenosis group compared to the control group (*p* < 0.001), indicating its potential as a superior diagnostic index for early IICAS detection using carotid Doppler ultrasound methods.
  - Barnawi et al.’s study [8] proposed a simple and efficient approach for recognizing normal and abnormal phonocardiogram (PCG) signals using Physionet data. The method utilizes data selection techniques like kernel density estimation (KDE) for signal duration extraction, signal-to-noise ratio (SNR), and Gaussian mixture model (GMM) clustering. The authors enhance the performance of 17 pre-trained Keras CNN models through these techniques. The results demonstrate excellent classification performance, achieving an overall accuracy of 97%, sensitivity of 94.6%, precision of 94.4%, and specificity of 94.6% by fine-tuning the VGG19 model after selecting the appropriate signal duration using KDE. This approach holds promise for developing accessible and user-friendly Cardiovascular disease recognition solutions, encouraging regular heart screenings for early detection.
  - Ribeiro et al. [9] published a literature review paper about the exploration of the infection mechanism, patient symptoms, and laboratory diagnosis regarding COVID-19. They also assess various technologies and computerized models, such as ECG, voice, and X-ray techniques, used for the accurate detection of COVID-19. The state-of-art literature reported high accuracy rates ranging from 85.70% to 100% for the diagnostic models. Based on these findings, they concluded that the existing models for COVID-19 detection have shown promising results, but there is still potential for improvement considering the diverse symptomatology and evolving understanding of the disease in individuals.
  - Battineni et al. [10] published a review paper focused on the use of ML models in the diagnosis of adult-onset dementia disorders. The authors explored the combination of ML algorithms with conventional magnetic resonance imaging (MRI) to enhance diagnostic accuracy. The findings indicate that ML techniques combined with MRI improve the diagnostic accuracy, with reported rates ranging from 73.3% to 99%. Alzheimer’s disease and vascular dementia were the most common adult-onset dementia disorders identified. The study concludes that ML should be integrated with conventional MRI techniques to achieve precise and early diagnosis of dementia disorders in older adults.
2. Personalized Medicine—ML techniques facilitate personalized medicine by leveraging patient-specific data to develop tailored treatment strategies. By considering individual characteristics, such as genetics, demographics, lifestyle, and medical history, algorithms can assist in predicting treatment outcomes and recommending optimal interventions [11]. This approach enables healthcare providers to deliver targeted therapies, optimize drug prescriptions, and minimize adverse effects, leading to improved patient outcomes and enhanced healthcare efficiency. This sub-area of study benefits from the contributions of the SI through the following research studies:
  - Kim et al. [12] used transfer transformers to identify drug–drug and chemical–protein interactions. They utilized the DDI Extraction-2013 Shared Task and BioCreative ChemProt datasets for extracting drug-related interactions. Two models were proposed: BERTGAT, incorporating a graph attention network for sentence structure, and T5slim_dec, adapting T5’s generation task for relation classification. T5slim_dec achieved remarkable performance with 91.15% accuracy on the DDI dataset and 94.29% accuracy for the CPR class group in ChemProt. However, BERTGAT did not significantly improve relation extraction. This highlights the language understanding capability of transformer-based approaches, which can comprehend language effectively without relying on additional structural information.
  - The study by Khadem et al. [13] addresses the challenge of accurate blood glucose prediction for diabetes management. They highlight the difficulty in determining the appropriate look-back window length, which affects the availability and relevance of information for decision-making. To overcome this challenge, the researchers propose an interconnected lag fusion framework using nested meta-learning analysis. They apply this framework to Ohio type 1 diabetes datasets and rigorously evaluate the models. The study demonstrates the effectiveness of their proposed method in personalized blood glucose level forecasting, providing valuable insights for informed decisions on insulin dosing, diet, and physical activity in diabetes management.
3. Public Health Surveillance and Outbreak Detection–ML plays a crucial role in public health surveillance systems by analyzing diverse data sources, including social media feeds, internet searches, electronic health records, environmental and bacteriological data [14]. By monitoring and detecting patterns, ML algorithms can identify potential disease outbreaks, track the spread of infectious diseases, and forecast disease trends. These insights enable public health authorities to allocate resources effectively, implement timely interventions, and prevent or mitigate the impact of epidemics. The SI makes a significant contribution to this particular sub-area of study through the inclusion of:
  - Rodrigues et al. [15] introduced a hybrid method combining pre-trained CNN keras models and classical ML models to visually discriminate different bacterial colonies based on their morphology on culture media. The system achieved high accuracy rates: 92% for Pseudomonas aeruginosa vs. Staphylococcus aureus, 91% for Escherichia coli vs. Staphylococcus aureus, and 84% for Escherichia coli vs. Pseudomonas aeruginosa.
4. Health Behavior Analysis and Intervention—ML algorithms can analyze large-scale health behavior data to identify risk factors, understand population health trends, and develop targeted interventions. By mining data from wearable devices, mobile apps, and social media platforms, ML models can provide insights into individuals’ behaviors, habits, and health outcomes [16]. This information can support the design of personalized interventions, health promotion campaigns, and policy recommendations, empowering individuals to make healthier choices and promoting population-level well-being. The SI actively contributes to this sub-area of study by including the following manuscripts:
  - Promsri et al. [17] studied the relationship between walking stability and fall risk markers in older adults. Three-dimensional lower-limb kinematic data from 43 healthy individuals were analyzed using principal component analysis (PCA) to extract principal movements (PMs) representing different components of walking. The largest Lyapunov exponent (LyE) was applied to the PMs as a measure of stability. Fall risk was assessed using the Short Physical Performance Battery (SPPB) and the Gait Subscale of Performance-Oriented Mobility Assessment (POMA-G). Results indicated a negative correlation (*p* ≤ 0.009) between SPPB and POMA-G scores and LyE in specific PMs, suggesting that increased walking instability is associated with higher fall risk.
  - Gupta et al. study [18] aimed to detect and address stress, which is a significant factor affecting mental health and overall well-being. In this study, a novel approach utilizing audio-visual data processing is proposed to detect human mental stress. By employing the cascaded RNN-LSTM strategy, the study achieved a high accuracy of 91% in classifying emotions and distinguishing between stressed and unstressed states using the RAVDESS dataset.
5. Healthcare Resource Optimization—ML can optimize healthcare resource allocation by predicting patient demand, improving scheduling and resource utilization, and optimizing healthcare facility operations. By analyzing historical data and considering factors such as patient demographics, disease prevalence, and resource availability, ML models can assist in optimizing bed occupancy, staff allocation, and healthcare supply chains [19]. This approach enhances operational efficiency, reduces costs, and improves patient access to timely and appropriate care. Within this sub-area of study, the SI offers the following valuable contribution:
  - da Silva et al. [20] proposed a methodology to analyze the performance of measurement systems during the design phase using the Monte Carlo method. The methodology was applied to a simulated ECG, estimating a measurement uncertainty of 3.54% with 95% confidence. The analysis revealed that the preamplifier module had a greater impact on the measurement results compared to the final stage module, suggesting that interventions in the preamplifier module would yield more significant improvements.

To conclude, ML has revolutionized health and public health decision support systems by enabling data-driven insights and informed decision-making. By harnessing the power of ML algorithms, healthcare professionals and public health authorities can improve disease diagnosis and prognosis, personalize treatment strategies, detect outbreaks, analyze health behaviors, and optimize resource allocation. As technology continues to advance, the integration of ML in health and public health applications will play an increasingly significant role in transforming healthcare delivery and improving population health outcomes.
